# A Study of Type II ɛ-PL Degrading Enzyme (pldII) in *Streptomyces albulus* through the CRISPRi System

**DOI:** 10.3390/ijms23126691

**Published:** 2022-06-15

**Authors:** Qinyu Li, Xiaojia Chen, Yuanjie Wu, Zheng Chen, Yang Han, Peng Zhou, Jiping Shi, Zhijun Zhao

**Affiliations:** 1 Lab of Biorefinery, Shanghai Advanced Research Institute, Chinese Academy of Sciences, No. 99 Haike Road, Shanghai 201210, China; liqy@shanghaitech.edu.cn (Q.L.); chenxj@shanghaitech.edu.cn (X.C.); 2 School of Life Science and Technology, ShanghaiTech University, Shanghai 201210, China; 3 University of Chinese Academy of Sciences, Beijing 100049, China; 4 Shanghai Institute of Pharmaceutical Industry, Gebaini Road 285, Shanghai 201203, China; wu.yuanjie@163.com; 5 School of Health Science and Engineering, University of Shanghai for Science and Technology, Shanghai 200093, China; 201570146@st.usst.edu.cn (Z.C.); 211310154@st.usst.edu.cn (P.Z.); 6 School of Forestry, Northeast Forestry University, Harbin 150040, China; hanyang0163@163.com

**Keywords:** ε-Poly-L-lysine, *Streptomyces albulus*, ε-PL degrading enzyme

## Abstract

ε-Poly-L-lysine (ε-PL) is a widely used antibacterial peptide polymerized of 25–35 L-lysine residues. The antibacterial effect of ε-PL is closely related to the polymerization degree. However, the mechanism of ε-PL degradation in *S. albulus* remains unclear. This study utilized the integrative plasmid pSET152-based CRISPRi system to transcriptionally repress the ε-PL degrading enzyme (pldII). The expression of *pldII* is regulated by changing the recognition site of dCas9. Through the ε-PL bacteriostatic experiments of repression strains, it was found that the repression of *pldII* improves the antibacterial effect of the ε-PL product. The consecutive MALDI-TOF-MS results confirmed that the molecular weight distribution of the ε-PL was changed after repression. The repression strain S1 showed a particular peak with a polymerization degree of 44, and other repression strains also generated ε-PL with a polymerization degree of over 40. Furthermore, the homology modeling and substrate docking of pldII, a typical endo-type metallopeptidase, were performed to resolve the degradation mechanism of ε-PL in *S. albulus*. The hydrolysis of ε-PL within pldII, initiated from the N-terminus by two amino acid-binding residues, Thr194 and Glu281, led to varying levels of polymerization of ε-PL.

## 1. Introduction

ε-Poly-L-lysine (ε-PL) is a bioantimicrobial amino acid polymer that consists of the α-carboxyl and ε-amino in L-lysine [1]. In general, ε-PL contains 25–35 L-lysine residues, has an isoelectric point of approximately 9.0, and does not have a significant secondary or tertiary structure [2]. ε-PL is a water-soluble peptide, stable at high temperatures and under acidic/alkaline conditions. With its broad spectrum of antibacterial effects, high efficiency, stability, and safety, ε-PL is widely used in the food and pharmaceutical industries [3].

Currently, ε-PL can only be generated by microbial fermentation. Several strains of bacteria secrete ε-PL, including *Streptomyces*, *Listeria monocytogenes*, and *Bacillus* [4,5,6]. In industry, ε-PL is typically synthesized by *Streptomyces albulus* [7]. ɛ-PL synthesis begins with glucose and the glycolytic tricarboxylic acid cycle pathway, which produces oxaloacetate; oxaloacetate then enters the diaminoheptanoic acid pathway to produce L-lysine, and lastly, ɛ-PL synthase (pls) on the cell membrane synthesizes ε-PL, which is secreted outside the cell [2,3,8,9,10]. In addition, within *S. albulus*, there are two degradative enzymes, ε-PL degrading enzyme (PLD) and type II ε-PL degrading enzyme (pldII), that hydrolyzed ε-PL [11,12,13]. PLD is identified as the exo-type peptidase that contains Zn^2+^ and is located on the cell membrane [14]. pldII was reported to be the major degrading enzyme of ε-PL in *S. albulus*, and it is translationally coupled to the position-adjacent *pls* gene. pldII is localized to the exterior of the cell membrane, and as an aminopeptidase with 51% amino sequence similarity to PLD, pldII has an opposite mode of action to PLD and is considered to be an endo-type aminopeptidase [15].

According to the previous studies, the antimicrobial activity of ɛ-PL depends on the size of its molecular weight, namely the degree of polymerization [16]. ɛ-PL with chain lengths of less than nine L-lysine residues exhibits little antimicrobial activity; when the number of L-lysine residues increases to 10, ɛ-PL begins to exhibit a profile of microbial growth inhibition [17]. Generally, ε-PL with a practical value ranges from 3.0 to 4.5 kDa, i.e., from 25–35 polymerized L-lysine residues [1]. It has been reported that ε-PL of the 15-27-mer exhibits a greater inhibitory activity against *E. coli*, while ε-PL of the 9-23-mer exhibits a greater inhibitory activity against *Saccharomyces cerevisiae* [18].

As the synthesis-degradation mechanism of ε-PL in industrial production strains has not been fully elucidated, it is difficult to precisely regulate the molecular weight of ε-PL in actual production. The ε-PL produced by microbial fermentation sometimes suffers from unstable antibacterial performances and low antibacterial potency. Kazuya Yamanaka et al. and Y. Hamano et al. found that the distribution range of ε-PL remained between 25 to 35 after inserting inactivated PLD and pldII in *S. albulus*, suggesting that the chain length of ε-PL could not be regulated through the expression of ε-PL degrading enzymes [11,13]. In the latest studies, it was found that mutating the two amino acids (Try646 and Leu883) at the junction site of the TM structural domain of ε-PL synthase (pls) produced ɛ-PL by fermentation with a change in chain length, and a degree of polymerization of 35 was identified as the initial chain length for ɛ-PL synthesis by *S. albulus* [18].

With the development of gene-editing tools that have matured regarding *Streptomyces*, further progress has been made in genetic engineering to breed strains that produce ε-PL. Delei xu et al. overexpressed the ammonium transporter protein gene *amtB* in *S. albulus* PD-1, increasing the ε-PL yield from 22.7 g/L to 35.7 g/L [19]. Aixia Wang et al. constructed a genetically engineered strain of *S. albulus* Q-PL2 by overexpressing *pls*, increasing ε-PL production efficiency by 88.2% ± 8.3% [20].

Since the rapid innovation of CRISPR/Cas9-based technologies, several effective CRISPR/Cas9-assisted gene manipulation tools have been developed in *Streptomyces* [21,22]. These tools are capable of carrying out a variety of gene-editing procedures, such as point mutations, gene deletions, and BGC deletions, in *Streptomyces* strains [23,24]. A CRISPR interference (CRISPRi) system that is derived from CRISPR/Cas9 and contains the nuclease-defective Cas9 nuclease (dCas9) coexpressed with sgRNA was also established in *Streptomyces* for efficient gene repression [25]. Yaojun Tong et al. developed a CRISPRi system based on a temperature-sensitive pSG5 backbone, in which the dCas9 gene was controlled by the thiostrepton inducible promoter (tipAp) to reversibly control the expression of the target gene [26]. Yawei Zhao et al. reported a new CRISPRi system based on the chromosomal integration vector pSET152, and the dCas9 gene and sgRNA were designed to be controlled by the strong constitutive promoters ermE* and j23119, respectively, enabling multiplex gene repression [27].

Based on previous studies, it was shown that pldII plays a major role in the degradation of ε-PL. Since the open reading frame of *pldII* follows *pls* on the *S. albulus* genome [28], it is speculated that these two functionally-opposing enzymes are closely related in their action.

In order to investigate the function of pldII in the production of ε-PL polymerization in *S. albulus*, this report utilizes the CRISPRi system for in situ transcriptional repression of *pldII* in *S. albulus* and regulates the intensity of *pldII* expression by altering the site of dCas9 action. We found that the polymerization degree of ε-PL was affected by transcriptional repression of *pldII* within the *S. albulus*. Further protein homology modeling and substrate docking of pldII protein were performed to analyze the degradation mechanism of ε-PL in *S. albulus*. This is the first report on the application of the CRISPRi system in *S.albulus*, as well as the first study demonstrating the function of the pldII in regulating the degree of polymerization of ε-PL.

## 2. Results and Discussion

### 2.1. Gene pldII Repression by the Integrative CRISPRi System in S. albulus

In this study, we investigated and analyzed the feature of pldII in the production of ε-PL in *S. albulus* by using the CRISPR/dCas9-mediated gene repression system reported by Yawei Zhao et al. [22] to repress the expression of the ε-PL degrading enzyme (pldII) in *S. albulus*.

**Figure 1:**
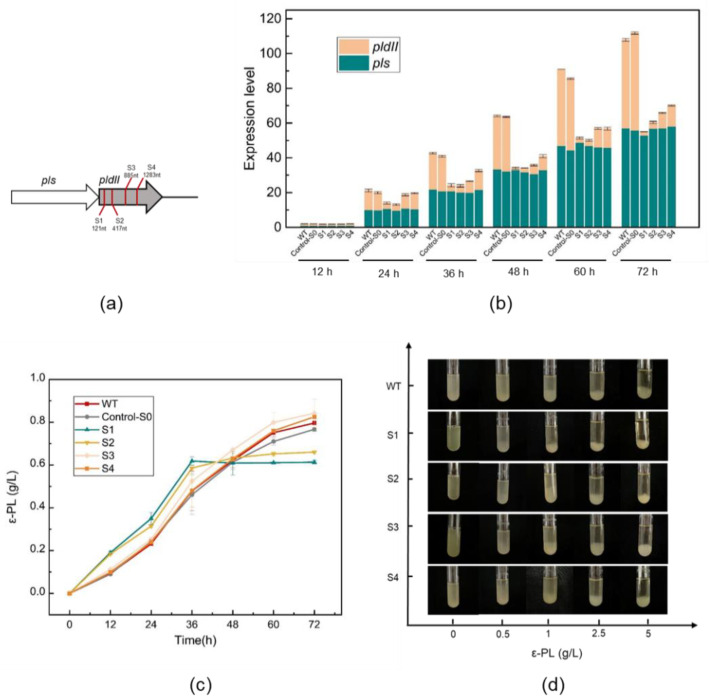
CRISPRi-mediated *pldII* repression in *S. albulus*. (**a**) Positions of four sgRNA protospacers on *pldII*; (**b**) Transcriptional analysis of *pldII* and *pls* in *S. albulus*; (**c**) Effect of *pldII* gene expression on the ε-PL production by *S. albulus*. (**d**) Effect of *pldII* gene expression on the minimum inhibitory concentration (MIC) for *S. albulus* against ε-PL. The data represent the means of three separate experiments, and the error bars represent the standard deviation. Some error bars cannot be seen due to small standard deviations.

The length of the open reading frame of the *pldII* gene is 1404 bp. pldII is located adjoining the ε-PL synthase (pls) on the genome. Figure 1a described the location of the target gene *pldII* on the genome and indicated the location and distribution of the four sgRNA protospacer sites (S1–S4) that were selected within the open reading frame of the gene sequence in this study.

Afterward, a 72 h shake flask fermentation was carried out with the constructed strain to initially verify its ability to produce ε-PL. During the fermentation of *S. albulus*, high concentrations of glucose and (NH_4_)_2_SO_4_ in the medium were essential to ensure the intensity of the strain growth and the intensity of ε-PL production by the strain [29]. In shake flask fermentation experiments, two controls were set up, a wild-type (WT) without genetic manipulation and a control-S0, in which the dcas9 plasmid was introduced without the sgRNA protospacers.

When conducting shake flask fermentation, we found that after repressing *pldII*, the repressed strains consumed glucose faster due to the additional expression of the CRISPRi system. After shaking the flask at 36 h, if the glucose content was not high enough, the pH increased and then destroyed the whole cultural system [30,31]. After further optimization, we finally decided to use an initial concentration of 50 g/L glucose and 25 g/L (NH_4_)_2_SO_4_ in the 72 h shake flask fermentation medium to guarantee the bacterial volume, and products accumulated normally. The results of ε-PL accumulation in shake flask fermentation for each strain are shown in Figure 1c. Figure 1c shows that the trend of ε-PL accumulation was largely unaffected in the strains with weaker repression of *pldII*, and even at the end of 72 h of fermentation, the strains S3 and S4 also provided better yields than the WT strain.

These findings can be verified by the qRT–PCR results shown in Figure 1b. All qRT–PCR runs were conducted with three biological and three technical replicates. The choice of the position of sgRNA protospacers affects the intensity of *pldII* expression. qRT–PCR indicated that the same gene was expressed differently at different growth stages of *S. albulus* as it underwent fermentation. The results we determined are in line with previous literature descriptions, in that the repressive effect is stronger when the repression site is closer to the front end of the gene [27]. After 24 h of fermentation, the repression started to become significant, with the greatest difference between the suppressor strains and WT appearing at 72 h. During the 72 h shake flask fermentation of all *S. albulus* strains, we observed that the expression of ε-PL synthase (pls) remained at a steadily increasing level. Moreover, in the WT and control-S0 strains, the expression of *pldII* was found to be convergent with that of pls. Since they are located next to each other on the genome, we speculate that the two are transcriptionally coupled.

Figure 1d shows the minimum inhibitory concentration (MIC) value for *S. albulus* against ε-PL. After activation, *S. albulus* was transferred to a final concentration of 0, 0.5, 1, 2.5, and 5 g/L LB liquid test tube. After 24 h of incubation at 30 °C and 220 rpm, the growth status of the strain was photographed and recorded. It revealed that the more repressed *pldII* was, the lower the MIC value of the strain against ε-PL. WT strains generally showed significant strain sedimentation and lysis at ε-PL concentrations of 1 g/L; however, the S1 strain exhibited a significant degradation trend at ε-PL concentrations of only 0.5 g/L in LB medium. This might provide an explanation for the stagnation of the ε-PL yield by strains S1 and S2 in the later stages of shake flask fermentation. At 36 h, the concentration of ε-PL that had accumulated in the medium was 0.5~0.7 g/L. Under these growth conditions, *pldII* in the repressed strains could not work properly against the damage of ε-PL on the cell membrane of the strains, and subsequently, the bacteria started to degrade. Therefore, in the later stages of the shake flask fermentation, even if the medium was full of nutrients, the strain was still unable to complete further ε-PL production and accumulation.

**Figure 2:**
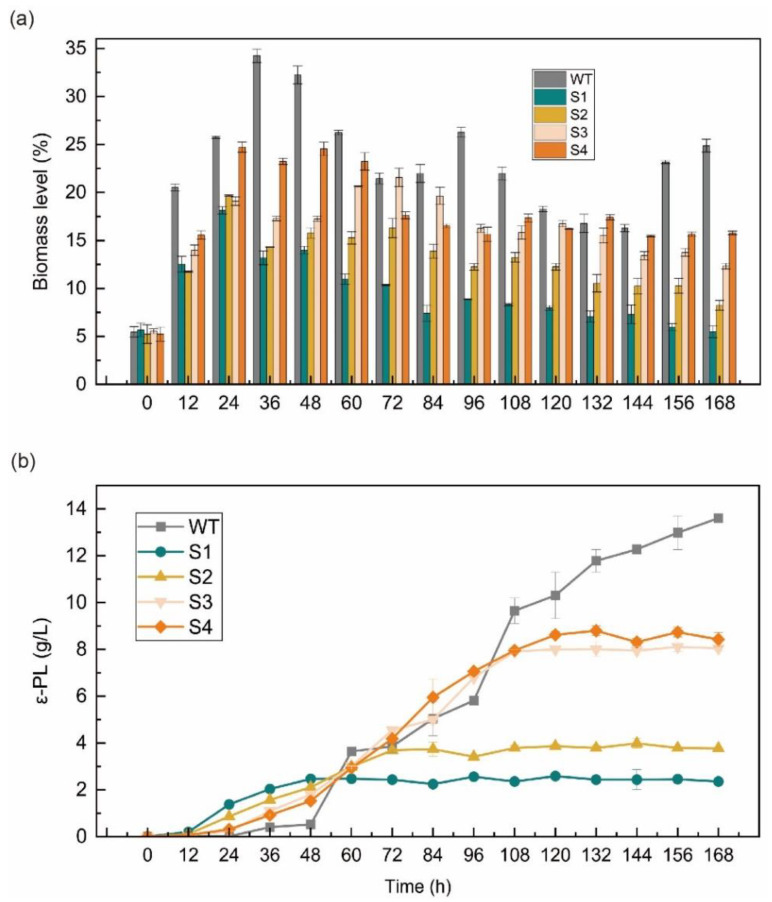
Biomass level and ε-PL production of the *S. albulus* strain under 168 h fed-batch fermentations. (**a**) Biomass level of the WT and four repressed strains; (**b**) ε-PL production of the WT and four repressed strains. The data represent the means of three samples per time interval, and the error bars represent the standard deviation. Some error bars cannot be seen due to small standard deviations.

Furthermore, we performed an additional fermenter fed-batch fermentation of the repressed strain (Figure 2). Dissolved oxygen was controlled at 30% while a 168 h 5 L fed-batch fermentation was performed, and the initial pH was 6.8 [32,33]. During the 168 h fed-batch fermentation, the pH was maintained above 6.0 with NH_3_·H_2_O from 0–36 h to ensure that the original accumulation of bacterial volume had occurred. After 36 h, the pH was controlled to 4.0 with NH_3_·H_2_O so that ε-PL could accumulate efficiently, and this was achieved by allowing the pH to fall freely as the strain consumed glucose. Since the control-S0 strain was similar to the WT strain in the transcriptional expression of synthesis and degradation genes regulating ε-PL (as shown in Figure 1b), only the WT strain was used as a control in the subsequent experiments.

In Figure 2a, a comparison of the amount of bacterial volume and the rate of glucose consumption for the repressed strains that were transformed in a 5 L fermenter is shown. In general, the accumulation of bacterial mass during *S. albulus* fermentation also fluctuated, with a rapid accumulation in the early stages and lysis in the middle and late stages as the pH decreased [34]. In a neutral environment, *S. albulus* grew vigorously and consumed glucose rapidly. Overall, the *pldII* repressed strains generally produced a lower accumulation of bacterial mass on the fermenters than that of the WT strain, which also generated a reaccumulation of bacterial mass at a later stage in a nutrient-rich environment. In contrast, the repressed strains were damaged by ε-PL in the fermentation broth, which prevented the reaccumulation of bacterial mass and gradually degraded the bacterial cells.

In addition, the accumulation of bacterial volume reflected the growth intensity, which was significantly stronger in the WT than in the repressed strains. At the beginning of fermentation (0~24 h), the strains exhibited differences in the bacterial mass level, but the growth intensity remained at a stable level. The growth intensity of the repressed strain was affected by the additional expression of dcas9 protein. After 24 h, the bacterial cells of repressed strains S1, S2, and S3 began to dissolve, while those of strain S4 remained at a relatively high level. When the CRISPRi system was active, part of the pldII function was preserved in the S4 strain with the sgRNA protospacers at the end of the pldII gene, indicating that the presence of pldII in *S. albulus* is necessary as a means of self-protection for the strain.

Figure 2b compares the ε-PL yields of these five strains. In general, ε-PL begins to accumulate after the pH value drops to 4.0 [35]. Since both pls and pldII prefer neutral pH levels, maintaining a pH of 4.0 in the middle and late stages of fermentation inhibits the activity of ε-PL degrading enzymes in the fermentation system [34]. During the 168 h fermentation of *S. albulus*, the combination of the higher biomass level and higher production of ε-PL was not necessary. If the bacterial mass was excessive, the reaction system was too viscous for the transfer of dissolved oxygen and nutrients; thus, the catalytic efficiency of the enzyme in the cells was affected [33]. However, after the repression of *pldII*, the time at which the fermentation system began to accumulate ε-PL was quickened from the usual 36 h to 24 h, and at 24 h, the ε-PL concentration was positively correlated with the degree of *pldII* repression. At 48 h, the pH of the fermentation system dropped freely to 4.0, and the WT strain began to accumulate ε-PL rapidly. However, the rate of ε-PL production by the S1 strain began to stagnate, and in combination with what was seen in Figure 1d and Figure 2a, at 48 h, the ε-PL concentration in the S1 fermentation system accumulated to 2.46 g/L, while the pH dropped and the bacterium began to dissolve rapidly; thus, the further accumulation of ε-PL could no longer occur. The other repressed strains also showed that ε-PL was unable to accumulate further at the late stage of fermentation, and this observation was related to the tolerance of the strains to ε-PL. The final ε-PL yields after 168 h of fermentation were 13.6 g/L, 2.35 g/L, 3.77 g/L, 8.04 g/L, and 8.42 g/L for the WT, S1, S2, S3, and S4 strains, respectively.

### 2.2. Analysis of ε-PL from the Repressed Strain

We purified ε-PL from the fermentation solution by performing multiple washes and filtering through a resin column. The antibacterial activity of ε-PL was measured using *Bacillus subtilis* 168 and *Escherichia coli* DH5α with an initial OD value of 0.5~0.65 as representative strains of Gram-positive and Gram-negative bacteria, respectively.

**Figure 3:**
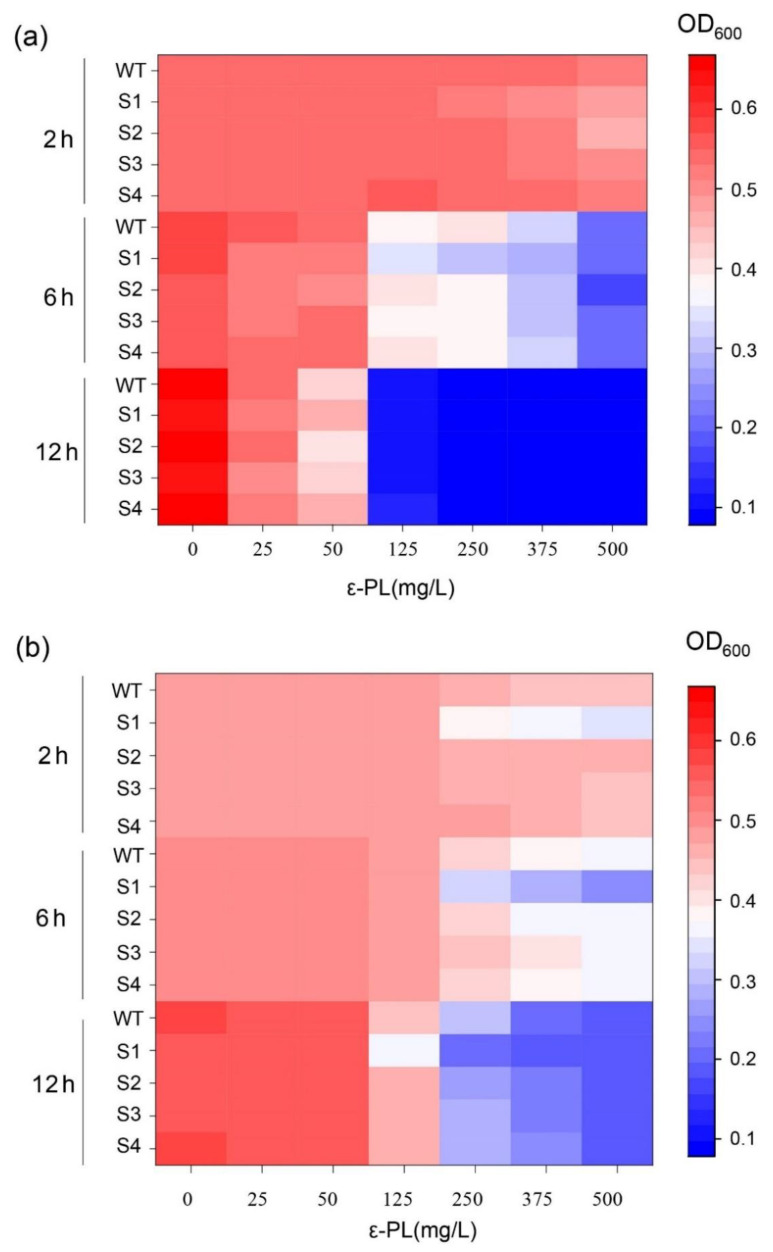
The antimicrobial activity of ε-PL from the WT and four repressed strains. (**a**) The antimicrobial activity of ε-PL against *B. subtilis* 168; (**b**) The antimicrobial activity of ε-PL against *E. coli* DH5α. The data represent the means of three samples per time interval, and the error bars represent the standard deviation. Some error bars cannot be seen due to small standard deviations.

The heatmap in Figure 3 demonstrates the changes in the OD values of *B. subtilis* 168 (Figure 3a) and *E. coli* DH5α (Figure 3b) over time at different final concentrations of 0, 25, 50, 125, 250, 375, and 500 mg/L ε-PL, which was extracted from the fermentation broths of different repression strains. The purified ε-PLs from the fermentation solutions of each strain were named WT, S1, S2, S3, and S4, along with their strain names. Overall, the inhibitory effect of ε-PL at low concentrations (0–50 mg/L) was not significant, and ε-PL was more likely to be effective against gram-positive bacteria than gram-negative bacteria. As shown in Figure 3a, the growth of *B. subtilis* 168 was significantly inhibited at 6 h of incubation and ε-PL at 125 mg/L. Among them, S1 showed a stronger antibacterial effect; the OD of *B. subtilis* 168 was only 0.3412 when incubated at 37 °C for 6 h with an LB medium that contained 125 mg/L S1 compared with the control OD of 0.57, and the OD was inhibited to 0.2288. When the incubation time was 12 h, the OD of *B. subtilis* 168 generally decreased below 0.1.

When testing the antibacterial effects of the ε-PL extracted from the repression strains against *E. coli* DH5α, we found that at concentrations greater than 250 mg/L, ε-PL more greatly inhibited the growth of *E. coli* DH5α compared to that of *B. subtilis* 168 with ε-PL in a concentration range of 125–500 mg/L. Interestingly, S1 also exhibited a higher antibacterial activity and resulted in significant inhibition of *E. coli* DH5a at 2 h of incubation. The OD of *E. coli* DH5α was only 0.1553 when LB medium that was supplemented with S1 at a final concentration of 500 mg/L, 37 °C, and 6 h were applied. Compared to the control OD of 0.4762, the growth intensity of *E. coli* was significantly inhibited by S1. The OD of *E. coli* DH5α in the blank control generally reached 0.54–0.55 with an incubation time up to 12 h, while the OD of *E. coli* DH5α was below 0.1 with 500 mg/L of WT, S1, S2, S3, and S4.

ε-PL is a compound that generally from 25–35 L-lysine polymerizations, and the magnitude of the inhibition effect of ε-PL is closely related to its chain length [36]. Based on the results of the repression experiments, it can be hypothesized that the repression of *pldII* using the CRISPRi system has a corresponding effect on the polymerization degree of the ε-PL synthesized by the strain. The molecular weight and the polymerization degree distribution of sample ε-PL (WT, S1, S2, S3, S4) were resolved by matrix-assisted laser-resolved ionization time-of-flight mass spectrometry (MALDI-TOF-MS). The degree of polymerization of ε-PL was calculated by dividing the molecular weight value by the relative molecular mass of the L-lysine residue. The basic principle of MALDI-TOF-MS is to disperse the sample in matrix molecules and form crystals [37]. When the crystals are irradiated with a laser, the matrix absorbs energy from the laser, the sample is desorbed, and the charge transfer between the matrix and the sample causes the sample molecules to ionize, which is detected by the proportion of the ion’s mass-to-charge ratio (M/Z) to the ion’s flight time and molecular weight. The accuracy of MALDI-TOF-MS is as high as 0.1~0.01% [38].

**Figure 4:**
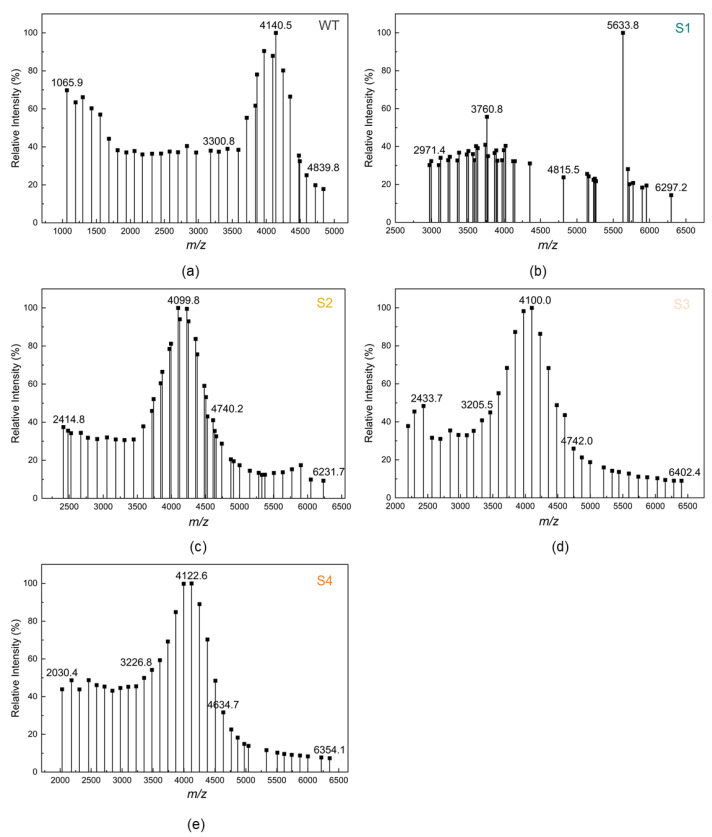
Matrix-assisted laser-resolved ionization time-of-flight mass spectrometry (MAL-DI-TOF-MS) analysis of the ε-PL produced by the *pldII* repressed strains. (**a**) ε-PL produced by WT strain; (**b**) ε-PL produced by S1 strain; (**c**) ε-PL produced by S2 strain; (**d**) ε-PL produced by S3 strain; (**e**) ε-PL produced by S4 strain.

The MALDI-TOF-MS results of 5 ε-PL samples (WT, S1, S2, S3, and S4) are shown in Figure 4. According to previous reports, ε-PL with chain length < 10 was considered to not inhibit bacteria [16], so we mainly focused on the peak emergence after lengths of 10. As shown in Figure 4, the distribution of ε-PL in the WT sample was in the range of molecular weight 1065.9–4839.8, which means that the ε-PL was polymerized from 9 to 38 L-lysine monomers. Two peaks of ε-PL appeared in sample S1 with molecular weights of 3760.8 and 5633.8, corresponding to the polymerization of 30 and 44 L-lysine monomers, respectively. The main molecular weight distributions of ε-PLs S2, S3, and S4 were 2414.8–4740.2, 2433.7–4742.0, and 2030.4–4634.7, corresponding to L-lysine polymerization degrees of 19–37, 19–37, and 16–36, respectively.

A small amount of ε-PL with a molecular weight greater than 5000 appeared in the ε-PL that was produced by the fermentation of the *pldII* repressed strains, which was not observed in the WT. Conversely, there was more small molecular weight ε-PL in the WT, which was not observed in the other samples. It is noteworthy that the samples WT, S2, S3, and S4 have similar molecular mass peaks, with the molecular mass of ε-PL ranging from 4099.8 to 4140.5, corresponding to the degree of polymerization of 32. However, the distribution of the degree of polymerization of ε-PL was a significant difference in sample S1, which had a concentrated distribution and a higher molecular weight. The particular peak polymerization degree of S1 was found at 44 and the distribution of the peak pattern was also different from the other samples. The results of MALDI-TOF-MS clarified the significant antibacterial effect of S1 in the bacteriostatic experiments. In strains with different repression intensities of *pldII* under the action of the CRISPRi system, the fermentation-produced ε-PL exhibited different molecular weight distributions.

### 2.3. Homology Modeling and Substrate Docking of pldII

**Figure 5:**
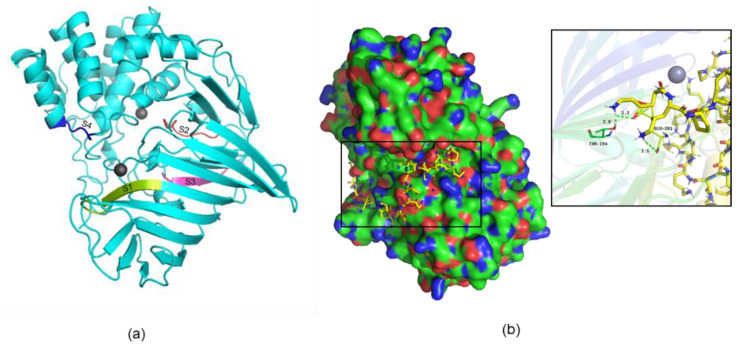
Homology modeling and substrate docking of pldII. (**a**) Homology structural model of pldII. The sites repressed in this study are marked in different colors; (**b**) pldII with a docked ε-PL. The diagram on the right shows the docking diagram with the active site.

To substantiate this study, we performed protein structure homology modeling of pldII (RMSD value: 0.245), and the results are shown in Figure 5a. The positions of the amino acid residues corresponding to the sites that were repressed in this study are marked in different colors in the figure, and the corresponding numbers are on the amino acid residues. pldII is a typical metalloendopeptidase, and the active region contains two Zn^2+^ ions, which play a role not only in the catalytic process of the enzyme but also in stabilizing the tertiary structure of pldII. We used Modeler10.1 software (Ben Webb, San Francisco, CA, USA) to perform multi-template homology modeling [39]. Like other reported metalloproteases, the conserved polypeptide segments form a large cavity that encloses the putative Zn-binding pocket and may confer specificity in the catalytic process [40]. The binding of the two Zn^2+^ ions was determined based on the conserved metal ion binding sites of the template protein, which in pldII are D44, T45, and D173, K174.

The results of docking of the protein model pldII with the substrate ε-PL are shown in Figure 5b. We modeled and optimized ε-PL using RDKit software [41], and docking was performed using AutoDock CrankPep software [42]. The lowest energy conformation in the construct was used. pldII is a typical peptide chain end-cleaving enzyme that sequentially hydrolyzes the peptide bond one by one starting from the N-terminus of ε-PL to produce free L-lysine. As shown in Figure 5b, the predicted active sites of pldII are Thr194 and Glu281. Therefore, we can further understand why the repression of pldII expression affects the chain length of ε-PL. In *S. albulus*, when the expression intensity of pldII was repressed, the ability of the cell to shear the N-terminal L-lysine in ε-PL one by one was diminished, resulting in the production of ε-PL monomers with different chain lengths. This critical hydrolysis ability of pldII is related to the enzyme active site, which is the binding site for Zn^2+^. Among the four repression sites selected in this study, the amino acid residue corresponding to S1 was located just at one of the Zn^2+^ action sites, which might be the reason why the S1 strain produced a significantly different ε-PL product.

## 3. Materials and Methods

### 3.1. Bacterial Strains, Plasmids, and Reagents

The strains and plasmids used in this study are listed in Table S1. *E. coli* DH5α was used for plasmid storage, and *E. coli* ET12567 was used as the parent strain for the inactivation experiment between *E. coli* and *S. albulus*. The control strain in this study was the laboratory-stored WT strain *S. albulus* WT, which was screened from soil that exhibited excellent ε-PL production. The strains constructed in this study were all derived from this wild-type strain.

The pSET-dCas9 plasmid for transcriptional repression of *pldII* in *S. albulus* was constructed according to the report of Yawei Zhao et al. [27]. The pSET-dCas9 plasmid was based on the chromosomal integration vector pSET152, and the dCas9 gene and sgRNA were designed to be controlled by the strong constitutive promoters ermE* and j23119, respectively. The primers used in this study are listed in Table S2.

*E. coli* was cultured in LB medium (10 g/L peptone, 10 g/L NaCl, 5 g/L yeast extracts, pH 7.0). *S. albulus* was cultured in MS medium (20 g/L soybean flour, 20 g/L mannitol, 20 g/L agar powder), and when intergeneric conjugation was performed with MS medium, an additional 10 mM MgCl2 was added to the medium.

The Taq DNA polymerase, restriction endonuclease, genome extraction kit, plasmid extraction kit, molecular purification kit, QuickCut™ Spe I, and QuickCut™ Hind III were purchased from Takara (Dalian, China). Recombinant plasmids were constructed by using the ClonExpress^®^ II one-step cloning kit, which was purchased from Vazyme Biotech Co. (Nanjing, China). Primers were synthesized by Sangon Biological Engineering Technology and Services (Shanghai, China).

### 3.2. Intergeneric Conjugation between E. coli and S. albulus

Due to the close proximity between *E. coli* and *S. albulus*, the shuttle integrative plasmid pSET152 was transferred from *E. coli* to *S. albulus*, leading to the intergeneric conjugation [43,44]. A single colony of *E. coli* ET12567 that contained the plasmid was picked the day before the conjugative transfer operation and was incubated in LB liquid medium containing 50 μg/mL kanamycin, 50 μg/mL chloramphenicol, and 50 μg/mL apramycin (Apr) at 37 °C with an overnight shaker at 220 rpm. A total of 500 µL of bacterial culture was transferred to shake flasks with 25 mL/250 mL LB medium and was incubated for approximately 4~6 h until the OD value was 0.4–0.6. The bacteria were collected by centrifugation at 4 °C and 8000 rpm in a 50 mL EP tube. Finally, the plasmid donor cells were obtained by washing twice with the same volume of LB medium to remove antibiotics.

The fresh slant of the *S. albulus* WT strain was inoculated into 50 mL/250 mL M3G liquid medium and incubated at 28 °C with shaking at 220 rpm for 16~20 h for initial activation. The activated strains were inoculated into M3G medium at 1% (*v*/*v*) and incubated for 16~20 h. The bacteria were collected by centrifugation at 4 °C and 8000 rpm. The cells of the host bacteria in the sensory state were obtained by washing twice with LB medium.

The plasmid donor bacteria and the host bacteria were mixed at a ratio of 1:1, and 200 µL of the mixture was evenly spread onto MS medium plates and incubated in a constant temperature incubator at 28 °C for 18~20 h. A total of 80~100 μg/mL Apr and 50 μg/mL nalidixic acid (Nal) were added after removal.

After the intergeneric conjugation operation, 2~3 days of incubation were performed at 30 °C, and when the conjugate grew on the medium, screening verification of the constructed strain was carried out. The single colony that was grown on the medium was verified by scratching it on MS medium with 80~100 μg/mL Apr to initially screen it out. After 2~3 days after incubation at 30 °C, when a single clone grew on the resistance plate, PCR validation was performed by passing the single colonies three times on MS medium. This step was to verify the transmission stability of the constructed strains. The PCR amplification program was initiated at 98 °C for 10 min, with 35 cycles of 98 °C for 30 s, 65 °C for 1 min, 72 °C for 30 s, and a final extension of 72 °C for 10 min.

### 3.3. Fermentation of S. albulus

The strain activation medium of *S. albulus* was the optimized M3G medium with the formula of 5% glucose, 0.5% yeast extract, 1% (NH_4_)_2_SO_4_, 0.1048% K_2_HPO_4_·3H_2_O, 0.136% KH_2_PO_4_, 0.07% MgSO_4_·7H_2_O, 0.0057% FeSO_4_·7H_2_O, 0.004% ZnSO_4_·7H_2_O, and an initial pH of 6.8. Glucose was sterilized separately. Approximately 1 mm^2^ of *S. albulus* spores were scraped from the plates into 250 mL triangular flasks containing 50 mL of LB medium and incubated for 12~16 h at 30 °C and 200 rpm.

The 72 h shake flask fermentation medium formulation for *S. albulus* was 5% glucose, 0.5% yeast extract, 1% (NH4)_2_SO_4_, 0.5% CaCO_3_, 0.1048% K_2_HPO_4_·3H_2_O, 0.136% KH_2_PO_4_, 0.07% MgSO_4_·7H_2_O, 0.0057% FeSO_4_·7H_2_O, 0.004% ZnSO_4_·7H_2_O, and a pH of 6.8. Glucose was sterilized separately. The fermentation conditions were 30 °C and 220 rpm.

The 168 h 5 L fed-batch fermentation medium formulation for *S. albulus* was 5% glucose, 0.5% yeast extract, 2.25% (NH_4_)_2_SO_4_, 0.056% K_2_HPO_4_·3H_2_O, 0.068% KH_2_PO_4_, 0.07% MgSO_4_·7H_2_O, 0.0057% FeSO_4_·7H_2_O, 0.004% ZnSO_4_·7H_2_O, and an initial pH of 6.8, in which glucose was sterilized separately. Inoculation was performed at a ratio of 5% (*v*/*v*), and the fermenter was filled with 2.5/5 L. The cultivation temperature was 30 °C, and the speed was coupled with dissolved oxygen, which was controlled at 30%.

### 3.4. Determination of ε-PL by HPLC

A total of 1.2 g of the ε-PL standard was weighed, dissolved in deionized water, and fixed to 1 L. Eight concentration gradients were diluted (1.2, 1.0, 0.8, 0.6, 0.5, 0.4, 0.2, 0). A standard curve was established using the peak areas displayed on the RID-10A/SPD-20A HPLC (SHIMADZU, Japan) at different concentrations of ε-PL.

The HPLC column was a C18 column, the mobile phase was 8% acetonitrile, the flow rate was 0.4 mL/min, the detection wavelength was 215 nm, the injection volume was 20 μL, and the temperature was 40 °C. The fermentation broth was collected and centrifuged at 12,000 r/min for 15 min.

The sample was prepared by diluting the fermentation broth at appropriate multiples in the range of the standard curve, and the concentration of ε-PL in the fermentation broth was calculated from the standard curve.

### 3.5. qRT–PCR Analysis

The total RNA of the five strains was extracted using the UNlQ-10 Column Trizol Total RNA (Sangon Biotech, Shanghai, China), and the total RNA was analyzed using 2X SG Fast qPCR Master Mix (Sangon Biotech, Shanghai, China) was used to obtain cDNA by reverse transcription, and a QuantStudio 3&5 Fluorescence PCR equipment (Thermo Fisher Scientific, Waltham, MA, USA) was used for real-time RT–PCR (qRT–PCR). The PCR conditions were 95 °C for 3 min, 95 °C for 5 s, and 60 °C for 30 s for 45 cycles. The RNA polymerase sigma factor gene (*hrdB*) was used as the internal reference gene, and the differential ploidy of ε-PL gene expression levels in the five strains was calculated according to the relative quantification of 2^−ΔΔCt^. The primer sequences used to amplify the target genes are shown in Table S2.

### 3.6. ε-PL Extraction

The 168 h fermentation broth was centrifuged at 8000 rpm for 30 min, and the supernatant was collected. NaOH was added to the supernatant to adjust the pH to 8.5, and the flocculent precipitate was removed by plate and frame filtration. The filtrate was first passed through a D155 resin column with a volume of 3 L at a flow rate of 0.8 BV/H (40 mL/min), then the resin column was rinsed with 4 BV, 0.2 N acetic acid at a flow rate of 1 BV/H (50 mL/min) to remove impurities, followed by a resin column rinsed with 12 BV, 0.1 N hydrochloric acid at a flow rate of 1 BV/H (50 mL/min) to elute ε-PL, and the eluate was collected. A 1× volume of activated carbon activated by the phosphoric acid method was added to the eluate, decolorized at 60 °C for 1 h, and the ε-PL was filtered out through medium speed filter paper. The volume of 711 resin was determined according to the concentration of chloride ions in the filtrate, and then the ε-PL filtrate was concentrated through a 711 resin column and nanofiltration membrane at a flow rate of 0.8 BV/H (40 mL/min). Finally, the water was evaporated in an oven to obtain the finished product of ε-PL.

### 3.7. MIC Determination of S. albulus against ε-PL

After 12–16 h of activation, 1 mL of *S. albulus* was transferred to a final concentration of 0, 0.5, 1, 2.5, or 5 g/L LB liquid test tube with 4 mL of liquid. After 24 h of incubation at 30 °C and 220 rpm, the growth status of the strain was photographed and recorded.

### 3.8. ε-PL Bacteriostatic Experiments

The ε-PL samples obtained from the fermentation broth of *S. albulus* WT and the repression strains S1, S2, S3, and S4 were used for the growth inhibition experiments with *Bacillus subtilis* 168 and *E. coli* DH5a.

The liquid medium was configured with different ε-PL final concentrations (125 mg/L, 200 mg/L, 250 mg/L, 375 mg/L, and 500 mg/L) based on LB medium. An overnight culture of *Bacillus subtilis* 168 and *E. coli* DH5a was added to 96-well plates containing the above medium, and the OD_600_ of the strain was adjusted to 0.55 to observe the change in its OD_600_. The OD_600_ values after 6 h of incubation at 37 °C were used to compare the inhibition potency of ε-PL from different sources to verify the inhibition effect of ε-PL. The OD_600_ of the medium in 96-well plates was measured in batch using a SynergyTM H1 Hybrid Multi-Mode Microplate Reader (BioTek, Winooski, VT, USA).

### 3.9. MALDI-TOF/TOF Analysis

An appropriate amount of ε-PL sample was dissolved by shaking with 50% acetonitrile aqueous solution (0.1% TFA). A total of 0.5 μL of sample solution was added to the 384-well MALDI target plate first and was dried naturally, then 0.5 μL of 0.5 g/L CHCA substrate solution was added (the solvent was a 50% acetonitrile aqueous solution containing 0.1% TFA) and the solution was dried naturally at room temperature. The samples were analyzed by mass spectrometry using rapifleX MALDI-TOF/TOF. The laser source was a three-dimensional Smartbeam Nd:YAG (355 mm) laser at 335 nm, the Smartbeam parameters were set to M5 small, and the spectra were acquired by accumulating 500 excitations at a laser frequency of 10 kHz with baseline cancellation. Data were acquired using positive ion reflection mode and automatic data acquisition mode. The instrument was first calibrated with equine myoglobin enzymatic peptides for external standard calibration. The primary mass spectra of the samples were scanned in the range of 200–10,000 Da. The obtained spectra were then analyzed and processed using the instrument software flexAnalysis.

### 3.10. Homology Modeling and Docking

Multiple template homology modeling was performed by sequence alignment using Modeller 10.1 software (Andrej Sali, San Francisco, CA, USA), and the binding of two Zn^2+^ ions was determined based on the conserved metal ion binding sites of the template protein. The ε-PL was modeled and optimized using RDKit software. The lowest energy conformation was subsequently adopted after docking using AutoDock CrankPep software (TSRI, San Diego, CA, USA).

## 4. Conclusions

To investigate the function of pldII in the production of the bioantimicrobial ε-PL in *S. albulus*, we used the CRISPRi system based on the shuttle integrative plasmid pSET152 to repress the transcription of *pldII* in *S. albulus* and to modulate the intensity of *pldII* expression by changing the site of dCas9 action. After the 72 h shake flask fermentation and the 168 h fed-batch fermentation, we found that inhibition of *pldII* expression caused ε-PL accumulation to begin at an earlier time in the fermentation system, but the final yield was not increased as expected. By measuring the MIC of the repressed strain against ε-PL, it was shown that the tolerance of the strain to ε-PL decreased significantly as the intensity of *pldII* repression in *S. albulus* increased. The fermentation yield of repressed strains was not enhanced due to the bacterial solubilization according to the accumulation of ε-PL in the fermentation system. pldII is a pivotal enzyme for self-protection in *S. albulus*. In a further investigation, we purified ε-PL from the fermentation broth of the repressed strain and verified its bacteriostatic activities against *B. subtilis* 168 and *E. coli*. DH5α. The results showed that ε-PL from strains with different repression strengths exhibited different bacteriostatic effects. Afterwards, a MALDI-TOF-MS analysis indicated that the polymerization degree distribution of ε-PL was changed after the repression of *pldII*. Compared to the previously reported degrees of polymerization of 25–35 L-lysine residues, the repression strains generated ε-PL with a polymerization degree of over 40. After further homology modeling and docking simulations, we considered that the typical endo-type metallopeptidase pldII affects the molecular weight of the ε-PL synthesized by *S. albulus* due to the function of the metallopeptidase, in which it shears peptide bonds starting from the N-terminus.

The CRISPRi system is a convenient and efficient genetic tool. We used the CRISPRi system to repress *pldII* and generated ε-PL products with different molecular weights, which have never been reported before. Moreover, presently reported studies on ε-PL were focused on optimizing the fermentation process to increase yield. The ε-PL antimicrobial effect is closely related to its molecular weight, but the mechanism of molecular weight regulation within the strain of *S. albulus* has been rarely reported. In this study, we obtained ε-PL with a significant antibacterial effect by regulating the expression of *pldII*, which laid the foundation for solving the problem of the unstable antibacterial potency exhibited by the ε-PL. In addition, this study validates the usability of the CRISPRi system in *S. albulus*, an *Actinomycete* with great scientific potential, for which the rapid and efficient CRISPRi tools can advance the function exploitation.

However, the CRISPRi tool causes metabolic stress when the bacterium grows due to the additional expression of dcas9 protein, which increases glucose consumption during fermentation and increases the production cost. With the repression of *pldII*, the defense mechanism of *S. albulus* was impaired, and high yields and high molecular weights could not be achieved simultaneously with the fermentation production of ε-PL. In future studies, a high yield of ε-PL could be achieved by domesticating and screening *S. albulus* strains that are highly tolerant by adding ε-PL to the medium.

## Data Availability

Not applicable.

## Supplementary Materials

The following supporting information can be downloaded at: https://www.mdpi.com/article/10.3390/ijms23126691/s1.
