# Supplementary material for: A Study of Type II ɛ-PL Degrading Enzyme (pldII) in Streptomyces albulus through the CRISPRi System
Source: Int J Mol Sci. 2022 Jun 15;23(12):6691. doi: 10.3390/ijms23126691 (PMC9223678; doi:10.3390/ijms23126691)
Supplement: Supplementary file 1 [file ijms-23-06691-s001.zip › ijms-1760962-supplementary.pdf]

Table S1. Strains and plasmids used in this study.

Name	Description	Source
**Strains**		
S. albulus WT	Wild type strain	Lab stock
E. coli DH5α	Host of plasmid	Lab stock
E. coli ET12567	Dam-13::Tn9 dcm-6 hsdM; harboring the non-transmissible RP4 derivative plasmid pUZ8002	Lab stock
**Plasmids**		
pSET-dCas9	Derived from pSET152, harboring the dCas9 expression cassette, including the ermE*p promoter, the dCas9 gene, and the fd terminator	[27]
pSET-dCas9-*pldII*-S1	Derived from pSET-dCas9, with the sgRNA cassette targeting the S1 site of *pldII*, j23119	This study
pSET-dCas9-*pldII*-S2	Derived from pSET-dCas9, with the sgRNA cassette targeting the S2 site of *pldII*, j23119	This study
pSET-dCas9-*pldII*-S3	Derived from pSET-dCas9, with the sgRNA cassette targeting the S3 site of *pldII*, j23119	This study
pSET-dCas9-*pldII*-S4	Derived from pSET-dCas9, with the sgRNA cassette targeting the S4 site of *pldII*, j23119	This study

Table S2. Primers used in this study.

Primer	Sequence(5′-3′)
pSET-dCas9-*pldII*-S1	CCTAGGTATAATACTAGT**CGAGACGGTGTCGTGGGCGTG**TTTTAGAGCTAGAAATAGCAAGT
pSET-dCas9-*pldII*-S2	CCTAGGTATAATACTAGT**CCGGTGCGGTCCCAGCCGATG**TTTTAGAGCTAGAAATAGCAAGT
pSET-dCas9-*pldII*-S3	CCTAGGTATAATACTAGT**GACCGGGTCTGGTTCTCGACG**TTTTAGAGCTAGAAATAGCAAGT
pSET-dCas9-*pldII*-S4	CCTAGGTATAATACTAGT**CGGTGTCGGCGCTGGCGCCGG**TTTTAGAGCTAGAAATAGCAAGT
pSET-dCas9-R	TATGACATGATTACGAATTCGG
dCas9-F	CAATCTGCATATGATGGACAAGAAGTACTCCATC
dCas9-R	TAGAATTCTCAGTCGCCGCCCAGCTGGGA
**Primers used in qRT-PCR**	
hrdB-RT-F	AGTTCGACTACACCAAGGGCTAC
hrdB-RT-R	TTGTTGATGACCTCGACCATGTG
pls-RT-F	CTCTTCCCGCTCTACGCC
pls-RT-R	GTGTCGTCGGCGAGGAAC
pldII-RT-F	GTTCGGCAGCAACCACATC
pldII-RT-R	AGCCCTCGTTGAGCCAGAT

Reference

27. Zhao, Y.; Zheng, G.; Jiang, W.; Deng, Z.; Wang, Z.; Lu, Y.J.B.J. CRISPR/dCas9-Mediated Multiplex Gene Repression in *Streptomyces*. *Biotechnol. J.* **2018**, *13*, e1800121. https://doi.org/10.1002/biot.201800121.
